# Health Perceptions, Multimorbidity, and New Fractures and Mortality Among Patients With a Fracture

**DOI:** 10.1001/jamanetworkopen.2024.8491

**Published:** 2024-04-24

**Authors:** Dunia Alarkawi, Thach S. Tran, Weiwen Chen, Lyn M. March, Fiona M. Blyth, Robert D. Blank, Dana Bliuc, Jacqueline R. Center

**Affiliations:** 1 Bone Epidemiology, Clinical and Translation Science Lab, Garvan Institute of Medical Research, University of New South Wales, Sydney, New South Wales, Australia; 2 Clinical School, Faculty of Medicine, St Vincent’s Hospital, University of New South Wales, Sydney, New South Wales, Australia; 3 Institute of Bone and Joint Research, Kolling Institute, Sydney, New South Wales, Australia; 4 Clinical School, Royal North Shore Hospital, St Leonards, New South Wales, Australia; 5 Clinical School, Concord Repatriation General Hospital, Sydney, New South Wales, Australia; 6 School of Population Health, Faculty of Medicine and Health, University of New South Wales, Sydney, New South Wales, Australia

## Abstract

**Importance:**

A high proportion of patients who sustain a fracture have multimorbidity. However, the association of multimorbidity with postfracture adverse outcomes, such as subsequent fractures and premature mortality, has not been widely explored.

**Objective:**

To examine the association of multimorbidity and self-rated health with subsequent fractures and mortality after fracture.

**Design, Setting, and Participants:**

This prospective cohort study included participants from New South Wales, Australia, in the Sax Institute’s 45 and Up Study (n = 267 357). Participants were recruited from July 2005 to December 2009 and followed up from the date of the incident fracture until subsequent fracture, death, or the end of the study (April 2017), whichever occurred first, with questionnaire data linked to hospital admission and medication records. Data analysis was reported between March and September 2023.

**Exposures:**

Charlson Comorbidity Index (CCI) score and self-rated health (SRH).

**Main Outcomes and Measures:**

The main outcomes were subsequent fracture or mortality after an incident fracture. Associations between SRH measures and subsequent fracture and mortality were also assessed. All analyses were stratified by sex given the different fracture and mortality risk profiles of females and males.

**Results:**

Of 25 280 adults who sustained incident fractures, 16 191 (64%) were female (mean [SD] age, 74 [12] years) and 9089 (36%) were male (mean [SD] age, 74 [13] years). During a median follow-up time of 2.8 years (IQR, 1.1-5.2 years), 2540 females (16%) and 1135 males (12%) sustained a subsequent fracture and 2281 females (14%) and 2140 males (24%) died without a subsequent fracture. Compared with a CCI score of less than 2, those with a CCI score of 2 to 3 had an increased risk of subsequent fracture (females: hazard ratio [HR], 1.16 [95% CI, 1.05-1.27]; males: HR, 1.25 [95% CI, 1.09-1.43]) and mortality (females: HR, 2.19 [95% CI, 1.99-2.40]; males: HR, 1.89 [95% CI, 1.71-2.09]). Those with a CCI score of 4 or greater had greater risks of subsequent fracture (females: HR, 1.33 [95% CI, 1.12-1.58]; males: HR, 1.48 [95% CI, 1.21-1.81]) and mortality (females: HR, 4.48 [95% CI, 3.97-5.06]; males: HR, 3.82 [95% CI 3.41-4.29]). Self-rated health was also significantly associated with subsequent fracture and mortality. Those reporting the poorest health and quality of life had the highest subsequent fracture risks, and their mortality risks were even higher.

**Conclusions and Relevance:**

In this cohort study, both CCI and SRH measures were associated with increased risk of subsequent fractures and mortality after fracture, underscoring the importance of managing the care of patients with comorbidities who sustain a fracture.

## Key Points

**Question:**

Are multimorbidity and self-rated health measures associated with subsequent fracture and mortality risk in adults aged 45 years or older with a fracture?

**Findings:**

In this cohort study of 25 280 adults with a fracture, multimorbidity and poor self-rated health were associated with higher risks of subsequent fractures and mortality.

**Meaning:**

The findings suggest the need to develop a practical framework for treating patients with multiple health conditions who sustain a fracture, who are usually underdiagnosed and undertreated.

## Teaser

This cohort study investigates associations of multimorbidity and self-related health measures with risk of subsequent fractures and mortality among patients aged 45 years or older with an incident fracture.

## Introduction

Fractures pose a significant public health issue with severe ramifications, including premature mortality,^1,2,3,4^ increased risk of subsequent fractures,^5,6^ disability, and a prolonged financial burden on the patient and the health care system.^7^ Like a fracture, multimorbidity, the coexistence of 2 or more chronic conditions in a patient,^8^ is age associated, with a prevalence in older adults ranging from 55% to 98%^9^ and rising with increasing life expectancy. Also like fractures, multimorbidity has been linked to several detrimental health outcomes, such as an increased likelihood of hospitalization, diminished quality of life, and disability.^10,11,12^

Several studies have reported a high proportion of patients with fractures and multimorbidity.^13,14,15^ A recent study by our group found that multimorbidity at the time of fracture significantly affects the gap in fracture management, reducing the likelihood of individuals receiving appropriate investigation and treatment for osteoporosis.^16^ However, the effect of multimorbidity on postfracture adverse outcomes has not been widely explored. The Charlson Comorbidity Index (CCI), an objective measure of comorbidity, has been shown to be associated with mortality, morbidity, and other health outcomes^17,18^ but has not been widely evaluated in people with fractures. Self-rated health (SRH), a subjective measure of health status, is another commonly used measure in clinical settings that has also been shown to be associated with mortality and other health outcomes independently.^19,20^ Like the CCI, SRH’s association with fracture risk and postfracture outcomes has not been examined, to our knowledge.

Therefore, we were interested in investigating the association of multimorbidity, measured through the CCI, the number of previous hospitalizations, and specific comorbidities, with the risks of subsequent fractures and mortality. Additionally, we investigated the association of SRH, including health status, quality of life, memory function, and quality of eyesight, with subsequent fractures and mortality.

## Methods

### Study Participants and Setting

**Figure 1.**
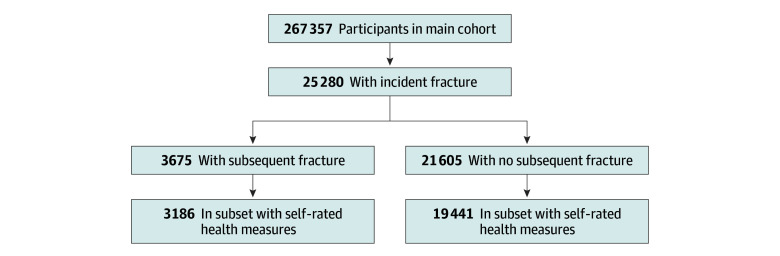
Flowchart of Study Participants

Participants in this cohort study were females and males from the Sax Institute’s 45 and Up Study. The 45 and Up Study is a population-based study that tracks 267 357 participants from New South Wales, Australia. The study, previously described in detail,^21^ invited individuals aged 45 years or older to participate by randomly selecting them from the Services Australia Medicare enrollment database, which encompasses most of the population. The study oversampled people aged 80 years or older and those living in rural or remote areas. Recruitment occurred from July 2005 to December 2009, and 19% of invited individuals participated after signing an informed consent form. They included approximately 11% of the New South Wales population aged 45 years or older who completed a survey and agreed to have their data linked to other administrative databases and be contacted for future follow-up surveys. The University of New South Wales Human Research Ethics Committee granted ethical approval for the 45 and Up Study, while the NSW Population & Health Services Research Ethics Committee approved the current study. This study followed the Strengthening the Reporting of Observational Studies in Epidemiology (STROBE) reporting guideline. Participants included those who sustained an incident low-trauma fracture after enrollment into the 45 and Up Study (Figure 1).

### Sources of Data

Baseline sociodemographic and clinical characteristics were obtained through self-administered questionnaires^22^ and administrative databases. The baseline questionnaire and a second questionnaire completed approximately 5 years later (between 2012 and 2015) provided information on health status, medical and surgical history, and factors associated with morbidity and mortality.

The administrative databases linked to the questionnaire data included the NSW Ministry of Health Emergency Department Data Collection (EDDC), containing information on emergency presentations; the NSW Ministry of Health Admitted Patient Data Collection (APDC), including information on all admissions to NSW public, private, and repatriation hospitals and private day procedure centers; the NSW Registry of Births, Deaths & Marriages (RBDM); and the Pharmaceutical Benefits Scheme (PBS), which contains data on drug prescriptions. The NSW Centre for Health Record Linkage linked all medical records using a probabilistic procedure, with an estimated false-positive rate of 0.5%. The Sax Institute facilitated the linkage to PBS data using a unique identifier and deterministic matching.

### Primary Outcome Measures

The primary outcomes were subsequent fracture or mortality after an incident fracture. Fractures were identified using diagnostic codes as outlined previously.^23^ These included *International Statistical Classification of Diseases and Related Health Problems, Tenth Revision (ICD-10)* codes from APDC data, Systematized Nomenclature of Medicine–Clinical Terms from EDDC data, and the Australian Classification of Health Interventions procedure codes. High-trauma fractures, multiple fractures involving 3 or more sites, pathological fractures, and fractures of the head, neck, fingers, and toes were excluded. The incident fracture was the first qualifying fracture that occurred after the recruitment date. Fractures were categorized as prior or subsequent based on their occurrence before the recruitment date or after the incident fracture, respectively. Information on mortality status was obtained from the RBDM.

### Demographic and Clinical Factors, Comorbidity, Multimorbidity, and SRH Measure Identification

Demographic and clinical factors analyzed were age at the time of fracture, weight, history of falls and fractures (before the incident fracture), aged care residency, needing help for disability (yes or no), and smoking status (yes or no). These variables were derived from the self-reported questionnaires. They were defined a priori based on their established significance as important factors associated with both fractures and mortality.

Individual comorbidities were identified from 3 sources: (1) the APDC data, which were based on *ICD-10* codes recorded within 5 years prior to the incident fracture; (2) the questionnaires, which provided information on specific comorbidities (including hypertension, rheumatoid arthritis, Parkinson disease, anxiety, and depression) for which the participants responded to the question, “Has a doctor ever told you that you have…?”; and (3) the Anatomical Therapeutic Chemical codes in the PBS data using the Rx-Risk index.^24,25^ The CCI was used to determine multimorbidity. The specific chronic conditions included in the CCI were identified from the same 3 aforementioned data sources. The CCI scores were classified into 3 groups based on the number of conditions: low multimorbidity (≤1), moderate multimorbidity (2-3), and severe multimorbidity (≥4). The number of hospitalizations in the 5 years before the incident fracture, as recorded by the APDC, was also used to measure multimorbidity.

The SRH measures were derived from the questionnaire and were completed by a subset of the participants with an incident low- or moderate-trauma fracture. They included overall health status, quality of life, overall memory function, and quality of eyesight. The SRH measures were ordinal class variables for which the participants responded to the question “In general, how would you rate your ….?” with either “excellent,” “very good,” “good,” “fair,” or “poor.”

### Statistical Analysis

All analyses were completed using SAS, version 9.4 (SAS Institute Inc). Baseline characteristics included self-reported information from the questionnaires on age, weight, history of falls in the 12 months before the incident fracture, history of fractures, aged care residency, need for help for disability, and smoking status. All analyses were stratified by sex given the different fracture and mortality risk profiles of females and males.

In this etiologic study, associations between multimorbidity and self-rated health and the risks of subsequent fracture and mortality after fracture were measured. Therefore, the associations between multimorbidity (assessed by the CCI score and the number of hospitalizations) and individual comorbidities and subsequent fracture and mortality were examined using cause-specific Cox proportional hazards regression models to estimate the simultaneous risk of subsequent fracture or mortality. This model is widely used in causal-inference studies and allows researchers to estimate the hazard ratios (HRs) of the outcomes of interest (in this study, subsequent fracture and mortality).^26,27^ The models in this study adjusted for the aforementioned a priori defined factors.

The association between SRH measures and subsequent fracture and mortality was assessed in the subset of participants who completed the questionnaires. Multivariate cause-specific Cox proportional hazards regression models that were adjusted for the same factors compared the HR of each stratum of the SRH measure with the reference stratum (participants who reported “excellent” for the question). Follow-up started from the date of the incident fracture and continued until subsequent fracture, death, or the end of the study (April 2017), whichever occurred first. Data were analyzed from March to September 2023.

## Results

### Baseline Characteristics

Of the 267 357 participants enrolled in the 45 and Up Study, 25 280 (9%) sustained incident fractures: 16 191 females (64%; mean [SD] age, 74 [12] years) and 9089 males (36%; mean [SD] age, 74 [13] years). Of those, 2540 females (16%) and 1135 males (12%) sustained a subsequent fracture, while 2281 females (14%) and 2140 males (24%) died without a subsequent fracture during a median follow-up time of 2.8 years (IQR, 1.1-5.2 years). Those who sustained a subsequent fracture were older, weighed less, were more likely to smoke, had experienced more prior falls and fractures, and were more likely to need help for disability. The CCI score and number of hospitalizations were higher for those in the group with subsequent fractures. A higher proportion of females with a subsequent fracture had an acute myocardial infarction, congestive heart failure, stroke, Parkinson disease, peptic ulcer, rheumatoid arthritis, and neurological conditions. Males with subsequent fractures were more likely to have congestive heart failure, cancer, Parkinson disease, plegia, rheumatoid arthritis, and depression (Table 1).

**Table 1.** Characteristics of Study Participants With Incident Fracture Stratified by Subsequent Fracture^a^

Characteristic	Females (n = 16 191)		Males (n = 9089)	
	Subsequent fracture (n = 2540)	No subsequent fracture (n = 13 651)	Subsequent fracture (n = 1135)	No subsequent fracture (n = 7954)
Age, mean (SD), y^b^	76 (12)^c^	72 (12)^c^	75 (13)^c^	73 (13)^c^
Weight, mean (SD), kg^d^	67 (16)^c^	69 (15)^c^	82 (17)^c^	83 (17)^c^
Falls^e^	769 (30)^c^	3383 (25)^c^	325 (29)^c^	1592 (20)^c^
Prior fracture^f^	286 (11)^c^	969 (7)^c^	102 (9)^c^	399 (5)^c^
Aged care residency^g^	90 (4)	405 (3)	34 (3)	239 (3)
Help for disability^h^	358 (14)^c^	1290 (9)^c^	133 (12)^c^	633 (8)^c^
Smoker^i^	165 (7)	886 (6)	105 (9)	691 (9)
Charlson Comorbidity Index score^j^				
≤1	1785 (70)^c^	10 178 (75)^c^	671 (59)^c^	5130 (65)^c^
2-3	606 (24)^c^	2708 (19)^c^	340 (30)^c^	2015 (25)^c^
≥4	149 (6)^c^	765 (6)^c^	124 (11)^c^	809 (10)^c^
Prior hospitalizations, No.^k^				
0	1794 (71)^c^	10 179 (75)^c^	650 (57)^c^	5023 (63)^c^
1-2	639 (25)^c^	2970 (22)^c^	369 (33)^c^	2302 (29)^c^
≥3	107 (4)^c^	502 (4)^c^	116 (10)^c^	629 (8)^c^
Comorbidities^l^				
Acute myocardial infarction	203 (8)^c^	729 (5)^c^	107 (9)	703 (9)
Congestive heart failure	458 (18)^c^	2132 (16)^c^	331 (29)^c^	1932 (24)^c^
Stroke	148 (6)^c^	546 (4)^c^	73 (6)	473 (6)
Diabetes	82 (3)	434 (3)	61 (5)	351 (4)
Chronic pulmonary disease	520 (20)	2721 (20)	184 (16)	1339 (17)
Kidney disease	55 (2)	283 (2)	50 (4)	295 (4)
Dementia	75 (3)	351 (3)	38 (3)	247 (3)
Cancer	462 (18)	2602 (19)	315 (28)^c^	1868 (23)^c^
Parkinson disease	45 (2)^c^	136 (1)^c^	37 (3)^c^	124 (2)^c^
Peripheral vascular disease	19 (1)	78 (1)	14 (1)	105 (1)
Peptic ulcer disease	459 (18)^c^	2219 (16)^c^	159 (14)	1145 (14)
Moderate-severe liver disease	13 (1)	74 (1)	8 (1)	57 (1)
Plegia	32 (1)	126 (1)	27 (2)^c^	119 (1)^c^
Rheumatoid arthritis	40 (2)^c^	88 (1)^c^	6 (1)^c^	21 (0.3)^c^
Neurological conditions	107 (4)^c^	381 (3)^c^	64 (6)	359 (5)
Depression	663 (26)	3423 (25)	244 (22)^c^	1473 (19)^c^

Abbreviation: *ICD-10, International Statistical Classification of Diseases and Related Health Problems, Tenth Revision*.

^a^ Data are presented as the number (percentage) of participants unless otherwise indicated.

^b^ 45 and Up Study question: “What is your date of birth?”^22^ Age at incident fracture was derived from this birth date.

^c^ Significant difference (2-sided *P* < .05) between those with subsequent fractures and those without.

^d^ 45 and Up Study question: “About how much do you weigh?”^22^

^e^ 45 and Up Study question: “During the past 12 months, how many times have you fallen to the floor or the ground?”^22^

^f^ 45 and Up Study question: “Have you had a broken/fractured bone in the last 5 years?”^22^

^g^ 45 and Up Study question: “What best describes your current housing? (1) house, (2) hostel for the aged, (3) nursing home, (4) flat, unit, apartment, (5) house on farm, (6) mobile home, (7) other retirement village, (8) self-care unit.”^22^

^h^ 45 and Up Study question: “Do you regularly need help with daily tasks because of long-term illness or disability?”^22^

^i^ 45 and Up Study question: “Are you a regular smoker now?”^22^

^k^ Admitted Patient Data Collection using *ICD-10* codes.

^l^ 45 and Up Study question: “Has a doctor ever told you that you have…”^22^ and Admitted Patient Data Collection using *ICD-10* codes and Anatomical Therapeutic Chemical codes in the Pharmaceutical Benefits Scheme data using the Rx-Risk Index.

In the subset with SRH measures (14 382 females [89%] and 8245 males [91%]), similar to the larger sample, the group with subsequent fractures was older, weighed less, had more prior falls and fractures, was more likely to need help for disability, and had a higher CCI score. In addition, those in the group with subsequent fractures reported poorer overall health and quality of life (Table 2).

**Table 2.** Characteristics of the Subset of Participants With Self-Rated Health Measures Stratified by Subsequent Fracture^a^

Characteristic	Females (n = 14 382)		Males (n = 8245)	
	Subsequent fracture (n = 2186)	No subsequent fracture (n = 12 196)	Subsequent fracture (n = 1000)	No subsequent fracture (n = 7245)
Age, mean (SD), y	75 (12)^b^	71 (12)^b^	75 (13)^b^	72 (13)^b^
Weight, mean (SD), kg	67 (16)^b^	69 (15)^b^	82 (17)^b^	83 (16)^b^
Falls	685 (31)^b^	3068 (25)^b^	293 (29)^b^	1471 (20)^b^
Prior fracture	236 (11)^b^	836 (7)^b^	90 (9)^b^	361 (5)^b^
Aged care residency	66 (3)	327 (3)	26 (3)	188 (3)
Help for disability	306 (14)^b^	1127 (9)^b^	120 (12)^b^	557 (8)^b^
Smoker	147 (7)	798 (7)	96 (10)	610 (8)
Charlson Comorbidity Index score				
≤1	1545 (71)^b^	9227 (76)^b^	598 (60)^b^	4738 (65)^b^
2-3	517 (24)^b^	2319 (19)^b^	290 (29)^b^	1805 (25)^b^
≥4	124 (6)^b^	650 (5)^b^	112 (11)^b^	702 (10)^b^
Overall health^c^				
Excellent	209 (10)^b^	1637 (13)^b^	87 (9)^b^	852 (12)^b^
Very good	605 (28)^b^	4070 (33)^b^	275 (28)^b^	2264 (31)^b^
Good	839 (38)^b^	4294 (35)^b^	364 (36)^b^	2658 (37)^b^
Fair	443 (20)^b^	1839 (15)^b^	228 (23)^b^	1226 (17)^b^
Poor	90 (4)^b^	356 (3)^b^	46 (5)^b^	245 (3)^b^
Quality of life^c^				
Excellent	339 (16)^b^	2531 (21)^b^	135 (13)^b^	1329 (18)^b^
Very good	633 (29)^b^	4223 (35)^b^	310 (31)^b^	2432 (34)^b^
Good	769 (35)^b^	3776 (31)^b^	342 (34)^b^	2348 (32)^b^
Fair	369 (17)^b^	1382 (11)^b^	178 (18)^b^	949 (13)^b^
Poor	76 (3)^b^	284 (2)^b^	35 (4)^b^	187 (3)^b^
Overall memory^c^				
Excellent	236 (11)^b^	1500 (12)^b^	97 (12)^b^	848 (12)^b^
Very good	521 (24)^b^	3542 (29)^b^	244 (24)^b^	1970 (27)^b^
Good	872 (40)^b^	4777 (39)^b^	348 (35)^b^	2698 (37)^b^
Fair	453 (21)^b^	2026 (17)^b^	251 (25)^b^	1417 (20)^b^
Poor	104 (5)^b^	351 (3)^b^	60 (6)^b^	312 (4)^b^
Quality of eyesight^c^				
Excellent	194 (9)	1157 (9)	89 (9)^b^	710 (10)^b^
Very good	568 (26)	3585 (29)	249 (25)^b^	2087 (29)^b^
Good	882 (40)	4995 (41)	431 (43)^b^	2989 (41)^b^
Fair	413 (19)	1976 (16)	193 (19)^b^	1207 (17)^b^
Poor	129 (6)	483 (4)	38 (4)^b^	252 (3)^b^

^a^ Data are presented as the number (percentage) of participants unless otherwise indicated.

^b^ Significant difference (2-sided *P* < .05) between those with subsequent fractures and those without.

^c^ 45 and Up Study question: “In general, how would you rate your overall health? Quality of life? Eyesight? Memory?”^22^

### Subsequent Fracture and Mortality Rates

During a median follow-up time of 2.9 years (IQR, 1.2-5.3 years) in females, 2540 (16%) sustained a subsequent fracture, which yielded a subsequent fracture rate of 5.2 per 100 person-years (95% CI, 5.0-5.4 per 100 person-years). A total of 2281 females (14%) died without a subsequent fracture, yielding a mortality rate of 4.6 per 100 person-years (95% CI, 4.5-4.8 per 100 person-years). In males, over a median follow-up time of 2.7 years (IQR, 1.0-5.1 years), 1135 (13%) sustained a subsequent fracture, yielding a subsequent fracture rate of 4.5 per 100 person-years (95% CI, 4.3-4.8 per 100 person-years), and 2140 (24%) died without a subsequent fracture, yielding a mortality rate of 7.9 per 100 person-years (95% CI, 7.6-8.2 per 100 person-years).

### Association Between Multimorbidity and Subsequent Fracture and Mortality

**Figure 2.**
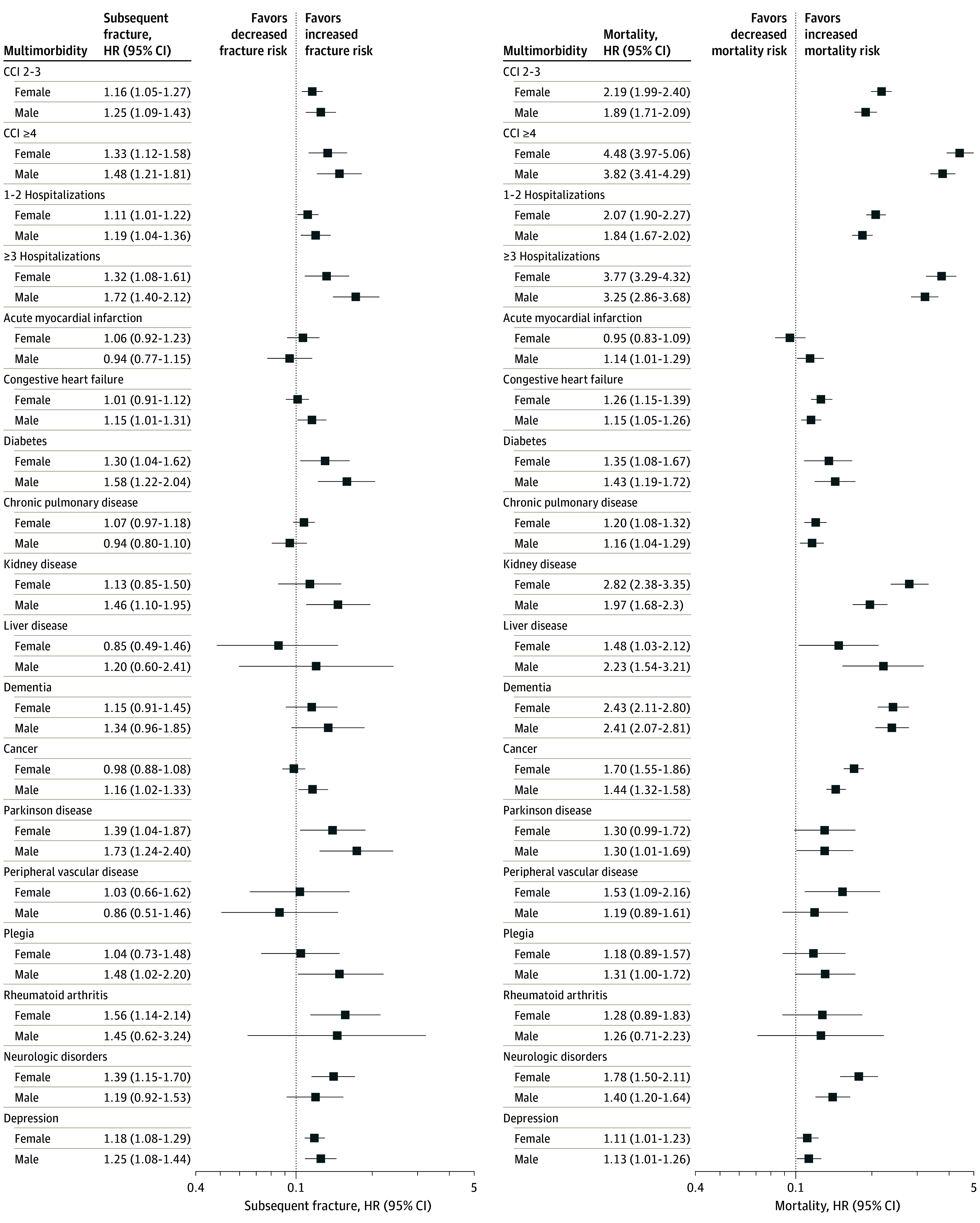
Forest Plots of Sex-Specific Hazard Ratios (HRs) for Subsequent Fracture and Mortality Risks After Fracture for Different Multimorbidity Measures Models were adjusted for age, weight, falls, prior fractures, aged care residency, help for disability, and smoking status. CCI indicates Charlson Comorbidity Index.

The cause-specific adjusted analysis demonstrated that in the female group, moderate and severe multimorbidity were associated with higher subsequent fracture risk (HR, 1.16 [95% CI, 1.05-.27] and 1.33 [95% CI, 1.12-1.58], respectively) and mortality risk (HR, 2.19 [95% CI, 1.99-2.40] and 4.48 [95% CI, 3.97-5.06], respectively) compared with lower multimorbidity. A similar pattern was observed in males, with moderate and severe multimorbidity being associated with higher subsequent fracture risk (HR, 1.25 [95% CI, 1.09-1.43] and 1.48 [95% CI, 1.21-1.81], respectively) and mortality risk (HR, 1.89 [95% CI, 1.71-2.09] and 3.82 [95% CI 3.41-4.29], respectively) compared with lower multimorbidity (Figure 2).

Similar trends in findings were reflected in the number of prior hospitalizations. Females with 1 to 2 or 3 or more hospitalizations had higher subsequent fracture risk (HR, 1.11 [95% CI, 1.01-1.22] and 1.32 [95% CI, 1.08-1.61], respectively) and mortality risk (HR, 2.07 [95% CI, 1.90-2.27] and 3.77 [95% CI, 3.29-4.32], respectively) than did those without hospitalizations. Males with 1 to 2 or 3 or more hospitalizations had higher subsequent fracture risk (HR, 1.19 [95% CI, 1.04-1.36] and 1.72 [95% CI, 1.40-2.12], respectively) and mortality risk (HR, 1.84 [95% CI, 1.67-2.02] and 3.25 [95% CI, 2.86-3.68], respectively) than did those without prior hospitalizations (Figure 2).

### Association Between Individual Comorbidities and Subsequent Fracture and Mortality

Specific comorbidities were significantly associated with subsequent fracture and mortality. In females, those with diabetes, Parkinson disease, rheumatoid arthritis, neurological disorders, and depression had higher subsequent fracture risk. In addition to the those comorbidities, congestive heart failure, chronic pulmonary disease, kidney disease, liver disease, dementia, cancer, and peripheral vascular disease were significantly associated with higher mortality risk (Figure 2).

More comorbidities were associated with subsequent fracture and mortality in males than in females. In males, diabetes, kidney disease, cancer, Parkinson disease, plegia, and depression were associated with higher subsequent fracture risk. In addition to the those comorbidities, acute myocardial infarction, chronic pulmonary disease, liver disease, dementia, and neurological conditions were associated with higher mortality risk (Figure 2).

### Association Between Self-Rated Health Measures and Subsequent Fracture and Mortality

The adjusted analysis demonstrated a gradual increase in subsequent fracture risk observed with SRH measures in both sexes. While not all estimates were significant, overall health and quality of life were significantly associated with subsequent fracture in both females and males and memory function only in females, especially in those who rated their health as poor in those measures (females: overall health HR, 1.54 [95% CI, 1.20-1.98]; quality of life HR, 1.58 [95% CI, 1.22-2.03]; and overall memory HR, 1.40 [95% CI, 1.11-1.77]; males: overall health HR, 1.49 [95% CI, 1.03-2.14]; quality of life HR, 1.37 [95% CI, 1.01-2.11]; and overall memory HR, 1.34 [95% CI, 0.98-1.83]) (Table 3). A similar trend was observed with mortality risk, but risk was higher in both females and males and across all health measures. Those who reported the poorest overall SRH had the highest mortality risk (females: HR, 3.99 [95% CI, 3.01-5.30]; males: HR, 3.21 [95% CI, 2.43-4.24]). For quality of life, among females with the poorest rating, the HR was 3.07 (95% CI, 2.37-3.97), while males had an HR of 2.51 (95% CI, 1.91-3.29) (Table 3). Additionally, females with the poorest memory function had the highest mortality risk (HR, 1.49 [95% CI, 1.19-1.86]), while males had an HR of 1.43 (95% CI, 1.15-1.78). Similarly, females with the poorest quality of eyesight had the highest mortality risk (HR, 1.33 [95% CI, 1.06-1.68]), whereas males had an HR of 1.65 (95% CI, 1.29-2.11) (Table 3).

**Table 3.** Association Between Different Strata of Self-Rated Health Measures and Subsequent Fracture and Mortality After Fracture

Health measure	HR (95% CI)^a^			
	Females		Males	
	Subsequent fracture risk	Mortality risk	Subsequent fracture risk	Mortality risk
**Overall health**				
Excellent	1 [Reference]	1 [Reference]	1 [Reference]	1 [Reference]
Very good	1.00 (0.86-1.17)	1.46 (1.14-1.87)^b^	1.08 (0.86-1.37)	1.29 (1.03-1.62)^b^
Good	1.20 (1.03-1.40)^b^	1.98 (1.56-2.50)^b^	1.17 (0.89-1.40)	1.63 (1.31-2.02)^b^
Fair	1.37 (1.16-1.62)^b^	2.86 (2.24-3.65)^b^	1.36 (1.06-1.74)^b^	2.32 (1.86-2.90)^b^
Poor	1.54 (1.20-1.98)^b^	3.99 (3.01-5.30)^b^	1.49 (1.03-2.14)^b^	3.21 (2.43-4.24)^b^
**Quality of life**				
Excellent	1 [Reference]	1 [Reference]	1 [Reference]	1 [Reference]
Very good	0.99 (0.87-1.13)	1.41 (1.16-1.71)^b^	1.12 (0.92-1.36)	1.47 (1.22-1.76)^b^
Good	1.18 (1.04-1.35)^b^	1.82 (1.51-2.19)^b^	1.14 (0.94-1.39)	1.81 (1.51-2.16)^b^
Fair	1.48 (1.27-1.72)^b^	2.30 (1.88-2.81)^b^	1.30 (1.03-1.63)^b^	2.05 (1.69-2.48)^b^
Poor	1.58 (1.22-2.03)^b^	3.07 (2.37-3.97)^b^	1.37 (1.01-2.11)^b^	2.51 (1.91-3.29)^b^
**Overall memory**				
Excellent	1 [Reference]	1 [Reference]	1 [Reference]	1 [Reference]
Very good	0.95 (0.82-1.11)	0.98 (0.82-1.16)	1.04 (0.82-1.30)	0.92 (0.77-1.10)
Good	1.13 (0.98-1.30)	1.04 (0.89-1.22)	1.01 (0.81-1.26)	1.03 (0.87-1.21)
Fair	1.24 (1.06-1.44)^b^	1.21 (1.02-1.43)^b^	1.25 (0.99-1.57)	1.17 (0.99-1.38)
Poor	1.40 (1.11-1.77)^b^	1.49 (1.19-1.86)^b^	1.34 (0.98-1.83)	1.43 (1.15-1.78)^b^
**Quality of eyesight**				
Excellent	1 [Reference]	1 [Reference]	1 [Reference]	1 [Reference]
Very good	0.96 (0.82-1.12)	1.06 (0.87-1.28)	0.91 (0.72-1.15)	1.09 (0.90-1.33)
Good	0.99 (0.85-1.16)	1.26 (1.05-1.52)^b^	1.00 (0.80-1.24)	1.30 (1.08-1.55)^b^
Fair	1.07 (0.90-1.26)	1.35 (1.11-1.64)^b^	1.04 (0.81-1.32)	1.35 (1.11-1.64)^b^
Poor	1.13 (0.91-1.41)	1.33 (1.06-1.68)^b^	0.91 (0.63-1.32)	1.65 (1.29-2.11)^b^

Abbreviation: HR, hazard ratio.

^a^ Models were adjusted for age, weight, falls, prior fractures, aged care residency, help for disability, and smoking status. In all models, each stratum’s HR was compared with the reference stratum (those who reported “excellent”) for that measure.

^b^ Significant at 2-sided *P* < .05.

## Discussion

This cohort study explored the association of multimorbidity with subsequent fracture and mortality after fracture and the association of self-rated health measures with those outcomes. We found that patients with a fracture and multimorbidity were more likely to sustain a subsequent fracture or die prematurely. The findings demonstrated that females with moderate (CCI score 2-3) or severe (CCI score ≥4) multimorbidity had significantly higher subsequent fracture risk and mortality risk compared with those with low multimorbidity (CCI score ≤1). Similar findings were observed in males, with the groups with moderate or severe multimorbidity having significantly higher risk of subsequent fracture and mortality than the group with lower multimorbidity. We also found a similar association with the number of prior hospitalizations in both females and males.

Moreover, we observed a gradual increase in the risk of subsequent fractures and mortality associated with SRH. In females, those with the poorest overall health and quality of life had a higher risk of subsequent fractures, while in males, the risk was elevated but to a lesser extent. The association of all health measures, including memory and eyesight quality, with mortality was statistically significant; both females and males with poor ratings for all 4 measures had the highest mortality risk.

While there is evidence of an association of individual comorbidities with increased initial fracture risk, limited work has been done addressing fracture and postfracture outcomes and multimorbidity per se. Diabetes,^28,29^ rheumatoid arthritis,^30^ Parkinson disease, and depression^31,32^ have all been associated with an increased risk of fractures. This study suggests that those comorbidities are also associated with an increased risk of subsequent fractures after an initial fracture. Furthermore, congestive heart failure, chronic obstructive pulmonary disease, kidney and liver diseases, dementia, and cancer were associated with increased mortality after fracture. These findings and the significant association of mortality with multimorbidity align with recent findings by our group in a Danish population.^33^ That study demonstrated that multimorbidity clusters defined by latent class analysis of individual comorbidities showed compounding of postfracture mortality in the high multimorbidity clusters beyond that attributable to multimorbidity or fracture alone.

Multiple studies have established an association between self-reported health and all-cause mortality.^34,35,36^ Fractures, particularly hip fractures, can considerably impact health and quality of life, leading to dependency.^37,38^ However, the potential association between SRH measures and subsequent fracture and mortality risks has received little attention. In this study, we observed that patients who rated their health as poor before sustaining a fracture were significantly more likely to have higher subsequent fracture and mortality risks than were those who reported better overall health and quality of life.

Additionally, among female patients who had sustained a fracture, lower memory function was associated with increased subsequent fracture and mortality risks. There was no association among males. This finding for females aligns with another study reporting an association between self-perceived memory loss and a higher risk of hip fracture.^39^ Furthermore, we found that poorer eyesight reported by patients before fracture was associated with higher mortality risks in both sexes but not subsequent fracture risks. Notably, a recent meta-analysis^40^ investigating the association between visual impairment and mortality discovered that individuals with vision impairment had a significantly higher risk of all-cause mortality than did those with normal vision or mild vision impairment. The severity of vision impairment was directly proportional to the elevated mortality risk.^40^

This study revealed that the CCI score and SRH measures both exhibited associations with subsequent fracture and mortality. The present findings highlight the need to develop a practical framework for treating patients with multiple health conditions who sustain a fracture. This need has been underscored by the significant underdiagnosis and undermanagement of osteoporosis after fracture in these patients shown by our group’s recent work.^16^

### Strengths and Limitations

This study’s strengths include the sizeable cohort with data linked to multiple sources to enhance completeness. The comprehensive documentation of chronic conditions using various data sources to identify comorbidities provides the most accurate classification.^41^ This enabled a detailed examination of the association of multimorbidity with subsequent fracture and mortality risks. However, this study has limitations. The Sax Institute’s 45 and Up Study cohort is healthier and has a higher educational level than the general population and may not represent the general population. In addition, linked administrative databases are generally prone to data misclassification and inaccuracy. Therefore, fracture misclassification may have resulted in some high-trauma fractures being included or low-trauma fractures being excluded.

## Conclusions

This cohort study demonstrated a significant association of multimorbidity with subsequent fracture and mortality after fracture. Higher CCI score or number of prior hospitalizations was associated with greater risks. Self-rated health before the incident fracture was also associated with subsequent fracture and mortality, with those reporting poorer health and quality of life demonstrating greater mortality and more subsequent fractures.

## Notes

Supplement.
Data Sharing Statement
