# Supplementary material for: Health Perceptions, Multimorbidity, and New Fractures and Mortality Among Patients With a Fracture
Source: JAMA Netw Open. 2024 Apr 24;7(4):e248491. doi: 10.1001/jamanetworkopen.2024.8491 (PMC11043901; doi:10.1001/jamanetworkopen.2024.8491)
Supplement: Supplement. — Data Sharing Statement [file jamanetwopen-e248491-s001.pdf]

Data Sharing Statement

Alarkawi. Health Perceptions, Multimorbidity, and New Fractures and Mortality Among Patients With a Fracture. *JAMA Netw Open*. Published April 24, 2024.
doi:10.1001/jamanetworkopen.2024.8491

Data
Data available: No

Additional Information
Explanation for why data not available: The data set could be available to other researchers if they obtain the necessary approvals. Further information on this process can be obtained from the 45 and Up Study (45andUp.research@ saxinstitute.org.au).
